# Care of Terminally ill Cancer Patients: Resource Allocation

**DOI:** 10.4103/0973-1075.73672

**Published:** 2010

**Authors:** Viroj Wiwanitkit

**Affiliations:** Professor, Wiwanitkit House, Bangkhae, Bangkok, Thailand 10160

Sir,

Editor, I read the recent publication by Bajwa *et al*, with a great interest.[1] Bajwa *et al*, concluded that “ICU care is the best form of treatment for terminally ill but resources should be used optimally so that a young deserving patient should not be sacrificed for the scarcity of resources”.[1] The question is not on the method of data collection but the conclusion. I would like to ask for the idea how that the young patient is specifically mentioned for the possible problem of resource allocation. There is no evidence from the study that might directly lead to this conclusion. Does this mean the present system of the ICU care has the problem on equal distribution of resource? Indeed, impact of cost, particularly in a managed care environment, seems to be an important thing to be concerned in dealing with cancerous patients whose death is thought to be imminent.[2] Nevertheless, care of the terminally ill patient is the common ethical problem and the poor distribution of the resource might be considered as a malpractice.[2]
